# Uso de glucómetros durante la prueba de tolerancia oral a la glucosa en niños para el diagnóstico de prediabetes y diabetes. Estudio comparativo

**DOI:** 10.1515/almed-2024-0017

**Published:** 2024-02-19

**Authors:** Blanca Fabre-Estremera, Estéfani Martínez-Chávez, Marta Manzano Ocaña, Atilano Carcavilla Urquí, María de los Ángeles Morales Sánchez, Inmaculada Pinilla Tejado, Isabel González-Casado, Itsaso Losantos García, Pilar Fernández-Calle, Antonio Buño Soto, Paloma Oliver

**Affiliations:** Servicio de Análisis Clínicos, Hospital Universitario La Paz, Madrid, España; Servicio de Endocrinología Pediátrica, Hospital Universitario La Paz, Madrid, España; Unidad de Bioestadística, Hospital Universitario La Paz, Madrid, España

**Keywords:** diabetes mellitus, prueba de tolerancia oral a la glucosa, pruebas a la cabecera del paciente

## Abstract

**Objetivos:**

A pesar de que las guías clínicas aún no recomiendan el uso de glucómetros en el lugar de asistencia al paciente (POCT) con fines diagnósticos, la prestación analítica de estos dispositivos ha mejorado significativamente. En este contexto, evaluamos la precisión analítica y la concordancia diagnóstica de los glucómetros POCT durante la prueba de tolerancia oral a la glucosa (PTOG), para el diagnóstico de prediabetes y diabetes en un estudio comparativo.

**Métodos:**

En este estudio prospectivo observacional, fueron reclutados pacientes pediátricos con indicación de PTOG, derivados a la Unidad de Diabetes entre diciembre de 2020 y septiembre de 2021. Durante la prueba funcional, se midió la glucemia en sangre venosa con dos glucómetros POCT (uno con conectividad y otro sin conectividad) y en el laboratorio central.

**Resultados:**

El estudio incluyó 98 pacientes. Observamos una elevada correlación entre los glucómetros y el laboratorio (coeficiente de Pearson=0,912 para el glucómetro sin conectividad y 0,950 para el glucómetro con conectividad). El tiempo de respuesta de la PTOG disminuyó significativamente (mediana glucómetro con conectividad: 2,02 horas [rango intercuartílico: 2,00–2,07], laboratorio: 11,63 horas [6,09–25,80]), con un coste global similar. La concordancia diagnóstica entre el glucómetro con conectividad y el laboratorio fue del 71,1 % (IC 95 % 61,5–79,2). La decisión clínica hubiera sido la misma en el 92,8 % de los casos, aunque no se habría indicado tratamiento en cuatro pacientes (4,1 %).

**Conclusiones:**

Durante las PTOG, los glucómetros POCT muestran una elevada correlación y una concordancia diagnóstica aceptable con el laboratorio, ofreciendo además el glucómetro con conectividad una reducción significativa del tiempo de respuesta, sin incrementar los costes. No obstante, dado que en algún caso podría haber un impacto clínico grave, los glucómetros POCT aún no deben ser utilizados con fines diagnósticos.

## Introducción

Una medición adecuada de la glucemia es esencial para el diagnóstico de la diabetes y la prediabetes [1]. La American Diabetes Association (ADA) define los criterios para diabetes y prediabetes en función de la concentración de glucosa y la presencia o ausencia de síntomas [1, 2]. La glucemia se puede medir en un laboratorio central o en el lugar de asistencia al paciente (POCT, por sus siglas en inglés *point-of-care testing*), mediante un conjunto de pruebas realizadas en el mismo lugar o cerca de donde se encuentra el paciente. Aunque la prestación analítica de los glucómetros POCT ha mejorado, su uso para fines diagnósticos todavía no está aceptado [1, 3]. Las plataformas POCT ofrecen diversas ventajas frente al laboratorio central, ya que, además de proporcionar los resultados de forma rápida, los dispositivos POCT precisan un menor volumen de muestra, lo que los hace especialmente atractivos para el manejo de pacientes pediátricos.

En relación con la prueba de tolerancia oral a la glucosa (PTOG), diversos estudios han analizado el nivel de concordancia diagnóstica entre los glucómetros y el laboratorio central [4], [5], [6]. Además de no haber incluido a poblaciones pediátricas, en los estudios publicados se utilizó sangre capilar en lugar de plasma venoso [4], [5], [6], como recomiendan las guías de práctica clínica de diabetes [1, 3]. Dada la escasa evidencia y discrepancias entre los resultados obtenidos, es necesario realizar más estudios [4], [5], [6].

Como hemos hecho en otros contextos [7], [8], [9], evaluamos la precisión analítica concordancia diagnóstica entre los glucómetros POCT durante la PTOG en pacientes pediátricos. Los objetivos del estudio fueron determinar la correlación a diferentes concentraciones de glucemia, la evolución de las mediciones de glucemia durante la PTOG, el tiempo de respuesta, la concordancia diagnóstica y los costes.

## Materiales y métodos

### Diseño del estudio y participantes

El laboratorio del Servicio de Análisis Clínicos del Hospital Universitario La Paz cuenta con la acreditación ISO 15189 y lidera desde hace 23 años una red de POCT multiparamétrica y multicéntrica acreditada según la ISO 22870.

Realizamos un estudio prospectivo observacional en pacientes pediátricos con indicación de PTOG atendidos en la Unidad de Diabetes del Servicio de Endocrinología Pediátrica entre diciembre de 2020 y septiembre de 2021. El estudio fue aprobado por el Comité de Ética de nuestro hospital (Código aprobación: 4.358). Todos los pacientes firmaron el consentimiento informado.

El tamaño muestral se calculó mediante la fórmula para estudios de equivalencia. Se estableció un error máximo de 4 mg/dL entre la concentración de glucosa (unidades SI, mmol/L; factor de conversión, 0,0555) obtenida en el laboratorio central y la obtenida con los glucómetros, con una varianza común para la glucosa de 20. Aplicando un nivel de significación de 0,05 y una potencia estadística de 0,90, se estimó un tamaño muestral de 81 pacientes.

**Figura 1:**
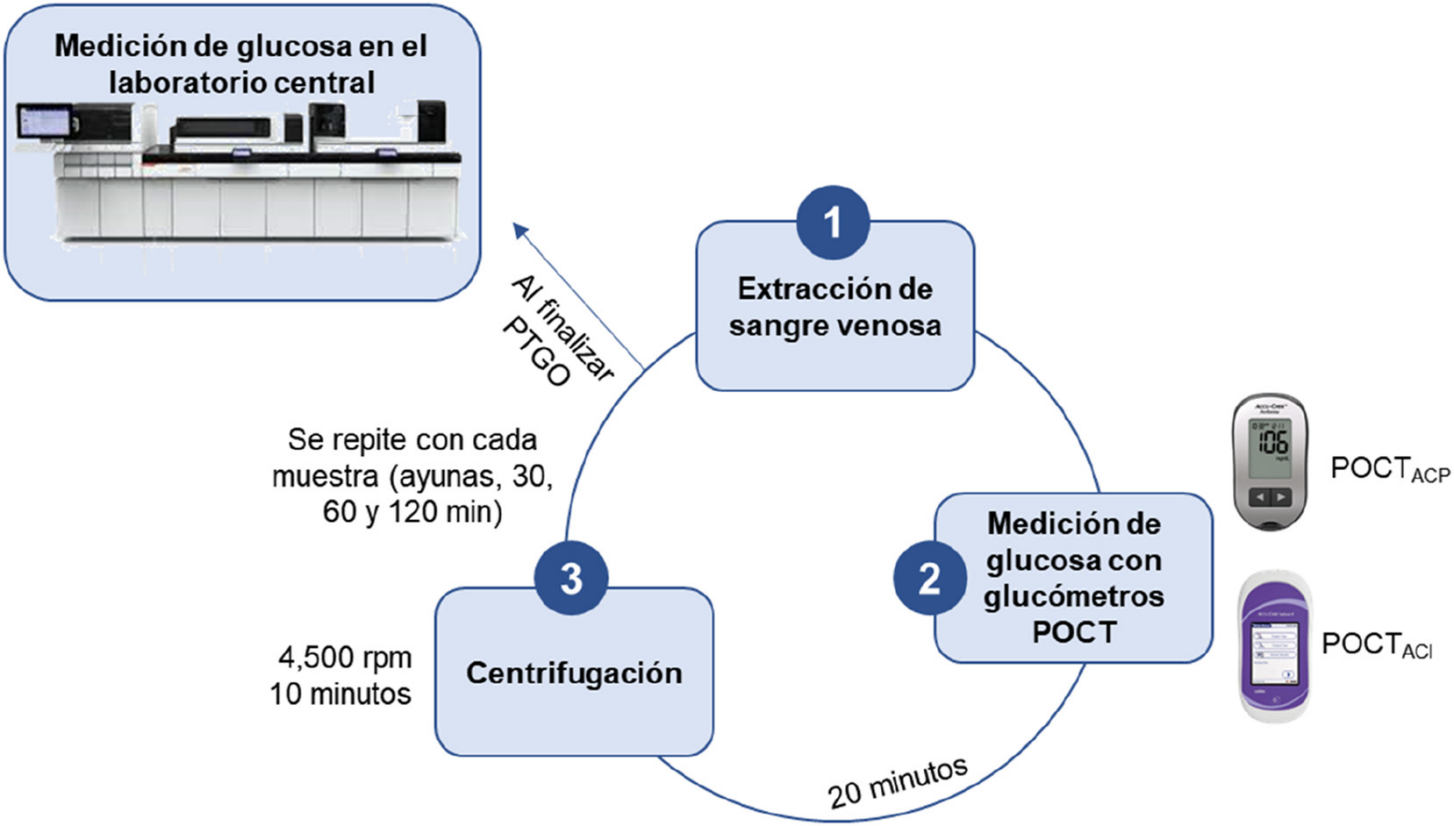
Medición de la glucosa durante el período de estudio. POCT_ACI_, glucómetro con conectividad; POCT_ACP_, glucómetro sin conectividad; PTOG, prueba de tolerancia oral a la glucosa.

Inmediatamente después de extraer la sangre venosa para la PTOG, se midió la glucosa en sangre total empleando dos glucómetros. Las muestras de sangre venosa se extrajeron en tubos con gel separador de suero (BD Vacutainer, Ciudad de México, México). Después de 20 min, las muestras se centrifugaron a 4.500 rpm durante 10 min en la Unidad de Diabetes y se almacenaron a temperatura ambiente. El proceso se repitió con cada extracción (en ayunas, a los 30, 60 y 120 min). Una vez finalizada la PTOG, las muestras se enviaron al laboratorio central para su análisis (Figura 1).

Para analizar la precisión analítica y concordancia diagnóstica de los glucómetros durante la PTOG, evaluamos dos glucómetros, ambos aprobados por la FDA para su uso profesional [10] y empleados habitualmente en nuestro hospital para la toma de decisiones clínicas, tomando como referencia los resultados del laboratorio central:

– Accu-Chek^®^ Performa (Roche Diagnostics, Basilea, Suiza): POCT_ACP_, sin conectividad, método enzimático (quinoproteína glucosa deshidrogenasa), muestras de sangre venosa (sangre total, factor de conversión automático a concentraciones en plasma [11]). Los coeficientes de variación interensayo (CV) fueron 2,25 % y 1,45 % del control interno de calidad (CIC), para una concentración media de 46 y 307 mg/dL, respectivamente.
– Accu-Chek^®^ Inform-II (Roche Diagnostics, Basilea, Suiza): POCT_ACI_, con conectividad, método enzimático (quinoproteína glucosa deshidrogenasa), muestras de sangre venosa (sangre total, factor de conversión automático a concentraciones en plasma [11]). Los CV fueron 1,75 % y 1,67 % del CIC, para una concentración media de 45 y 303 mg/dL, respectivamente.
– Atellica^®^ Solution-CH (Siemens Healthineers, Erlangen, Alemania): laboratorio central, método enzimático (glucosa hexoquinasa), muestras de sangre venosa (suero). Los CV fueron 1,96 % y 2,17 % del CIC, para una concentración media de 59 y 343 mg/dL, respectivamente. Actualmente es el que se utiliza para diagnóstico durante PTOG.

Los resultados obtenidos por el POCT_ACP_ se recogieron de forma manual en un registro, siguiendo la práctica clínica habitual. Los resultados obtenidos por el POCT_ACI_, así como por el laboratorio central se exportaron del sistema de información del laboratorio (LabTrak; Intersystems, Cambridge, Massachusetts, Estados Unidos).

### Correlación entre las mediciones de glucosa durante la PTOG

Previamente se realizó un estudio comparativo de los tres analizadores de glucosa basado en el procedimiento EP09 del Clinical and Laboratory Standards Institute [12]. Se procesaron tres muestras por duplicado (concentraciones de glucosa de entre 100 mg/dL y 222 mg/dL) en cada analizador durante tres días consecutivos, demostrando la intercambiabilidad de los resultados de los pacientes a los niveles de decisión clínica.

La correlación se evaluó en ayunas y a los 30, 60 y 120 min. Se realizó la comparación de métodos entre POCT_ACI_ y el laboratorio central.

### Tiempo de respuesta

Se definió el tiempo de respuesta (TDR) durante la PTOG como el tiempo transcurrido desde la primera extracción de sangre hasta el momento en el que el clínico tuvo acceso a la totalidad de los resultados.

### Concordancia diagnóstica

La concordancia diagnóstica entre los glucómetros y el laboratorio central (tomado como referencia) se evaluó siguiendo los criterios de la American Diabetes Association (ADA) (Tabla S1 del Material Suplementario) [1].

Las especificaciones de prestación analítica fueron basadas en variación biológica [13]. El error total (ET) se calculó mediante la siguiente ecuación [13]:

$$\text{ET}<1,65\times \left(0,5\times {\text{CV}}_{I}\right)+0,25\times {\left({\text{CV}}_{I}^{2}+{\text{CV}}_{G}^{2}\right)}^{1/2}$$

CV_I_: 4,9 %, CV_G_: 8,1 % [13].

El factor 0,5 hace referencia a una especificación deseable.

CV_I_, coeficiente de variación intraindividual; CV_G_, coeficiente de variación interindividual.

De este modo, se estableció un ET de 6,4 %, basado en especificaciones deseables que usamos en nuestro laboratorio. En caso de discrepancia en el diagnóstico entre el POCT_ACI_ y el laboratorio central, se calcularon las diferencias porcentuales, y se analizaron los casos que superaban dicha especificación.

### Costes

Los costes globales durante el periodo de estudio se evaluaron teniendo en cuenta los recursos materiales necesarios para cada analizador, mientras que los gastos de personal los proporcionó el Departamento de Recursos Humanos. Así mismo, se tuvieron en cuenta las horas de trabajo del personal por cada paciente. La información sobre el resto de la proporcionaron el Departamento de Compras y la Unidad de Servicios Administrativos.

### Conectividad

Dado que el POCT_ACP_ es un glucómetro sin conectividad y el POCT_ACI_ sí la tiene, se tuvieron en cuenta los errores de transcripción durante el periodo de estudio, para evaluar la fase postanalítica. Además, como objetivo exploratorio, el personal clínico rellenó un cuestionario de satisfacción sobre el dispositivo con conectividad (Archivo S1 del Material Suplementario).

### Análisis estadístico

Los datos cualitativos se presentan como frecuencias absolutas y porcentajes, mientras que los datos cuantitativos se expresan como medias y desviaciones estándar (DE) o como medianas y rangos intercuartílicos (RIC), dependiendo de la distribución de los datos.

La normalidad de las variables continuas se analizó con la prueba de Kolmogorov–Smirnov.

La asociación entre las concentraciones de glucosa se evaluó mediante el coeficiente de correlación de Pearson. Se utilizó la prueba t de Student y la prueba de Wilcoxon para analizar la asociación entre las concentraciones de glucosa y el TDR, respectivamente. La concordancia diagnóstica se analizó mediante el porcentaje de concordancia observado y sus intervalos de confianza al 95 % (IC 95 %).

Las pruebas estadísticas se consideraron bilaterales, mientras que aquellas con una probabilidad de error inferior al 5 % (p<0,05) se consideraron estadísticamente significativas. Se utilizó el programa SAS 9.3 (SAS Institute, Cary, Carolina del Norte, Estados Unidos).

## Resultados

Durante el periodo estudio, se indicó la prueba PTOG a 112 pacientes pediátricos. De éstos, siete padres y/o representantes rehusaron firmar el consentimiento informado. Excluimos a siete pacientes que no concluyeron la PTOG, obteniendo una muestra final de 98 participantes (Figura S1 del Material Suplementario). El 53,1 % eran niñas, con una mediana de edad (RIC) de 12 años [10], [11], [12], [13], [14].

### Correlación entre las determinaciones de glucosa durante la PTOG

En las se muestra la glucosa obtenida con los tres analizadores, así como los Tablas 1–2 coeficientes de correlación de Pearson. Observamos diferencias estadísticamente significativas entre las concentraciones medidas en ayunas, siendo los glucómetros los que mostraron concentraciones de glucosa más altas.

**Tabla 1:** Concentración de glucosa (mg/dL) durante la prueba de tolerancia a la glucosa.

	POCT_ACP_	POCT_ACI_	Laboratorio central
Ayuno	96 (8,3)^a^	96 (8,5)^a^	92 (9,0)
30 min	150 (26,5)	150 (28,6)	156 (28,6)
60 min	151 (37,3)	151 (37,1)	161 (45,9)
120 min	133 (33,0)	133 (32,3)	139 (36,9)

Media (DE); ^a^p<0,05. POCT_ACI_, glucómetro con conectividad; POCT_ACP_, glucómetro sin conectividad; DE, desviación estándar.

**Tabla 2:** Correlación de la glucemia entre los glucómetros POCT y el laboratorio central.

		POCT_ACP_	POCT_ACI_
		Coeficientes de correlación de Pearson	
Laboratorio central	Ayuno	0,787^a^	0,786^a^
	30 min	0,633^a^	0,937^a^
	60 min	0,878^a^	0,876^a^
	120 min	0,984^a^	0,980^a^
	Global	0,912^a^	0,950^a^

^a^p<0,001. POCT_ACI_, glucómetro con conectividad; POCT_ACP_, glucómetro sin conectividad.

En el archivo S2 del Material Suplementario se muestra la comparación de métodos entre POCT_ACI_ y el laboratorio central.

### Tiempo de respuesta

**Figura 2:**
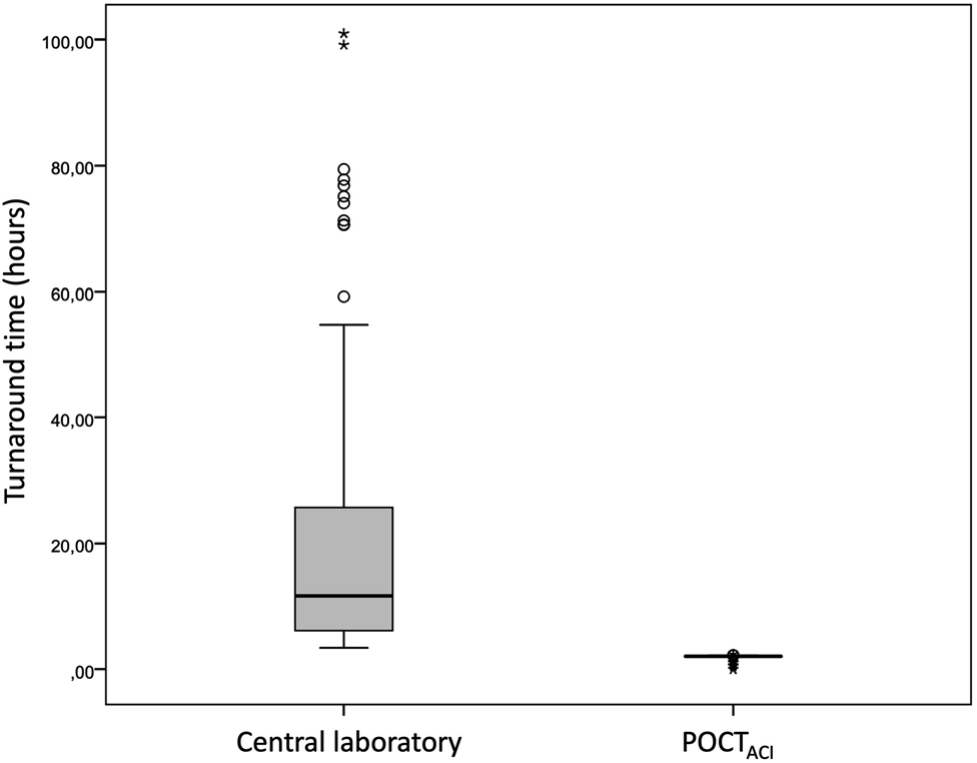
Tiempo de respuesta de la prueba de tolerancia oral a la glucosa utilizando glucómetros POCT vs. el laboratorio central. POCT_ACI_, glucómetro con conectividad.

Se observaron diferencias estadísticamente significativas en la mediana (RIC) del TDR para la PTOG entre el glucómetro con conectividad y el laboratorio central (2,02 horas (2,00–2,07) y 11,63 horas (6,09–25,80), respectivamente, p<0,001) (Figura 2). POCT_ACP_ tiene mayor TDR que POCT_ACI_ ya que hay que transcribir los resultados a la historia clínica electrónica de los pacientes durante la jornada laboral.

### Concordancia diagnóstica

La concordancia diagnóstica entre el laboratorio central y POCT_ACP_ fue del 74 % (IC 95 % 69,4–81,7) y entre el laboratorio central y POCT_ACI_ fue del 71,1 % (IC 95 % I 61,5–79,2) (Tabla 3).

**Tabla 3:** Concordancia diagnóstica entre los glucómetros POCT y el laboratorio central.

		Laboratorio central				
		Normal	AGA	ATG	AGA+ATG	Diabetes
POCT_ACP_, n, %	Normal	**44 (80 %)**		6 (28,6 %)		
	AGA	10 (18,2 %)	**5 (100 %)**	2 (9,5 %)	1 (12,5 %)	
	ATG	1 (1,8 %)		**11 (52,4 %)**	1 (12,5 %)	2 (28,6 %)
	AGA+ATG			2 (9,5 %)	**6 (75 %)**	
	Diabetes					**5 (71,4 %)**
POCT_ACI_, n, %	Normal	**44 (80 %)**	1 (20 %)	7 (33,3 %)		
	AGA	10 (18,2 %)	**4 (80 %)**	1 (4,8 %)	1 (12,5 %)	
	ATG	1 (1,8 %)		**12 (57,1 %)**	1 (12,5 %)	4 (50,0 %)
	AGA+ATG			1 (4,8 %)	**6 (75 %)**	1 (12,5 %)
	Diabetes					**3 (37,5 %)**

AGA, alteración de la glucemia en ayunas; ATG, alteración de la tolerancia de glucosa; POCT_ACI_, glucómetro con conectividad; POCT_ACP_, glucómetro sin conectividad. Negrita: concordancia diagnóstica de los glucómetros con respecto al laboratorio central (referencia).

En los casos discordantes, se calculó la diferencia porcentual de las concentraciones de glucosa entre los glucómetros y el laboratorio central. Observamos más casos (11 casos) de diferencias porcentuales inferiores a nuestra especificación (ET=6,4 %) con POCT_ACI_ que con POCT_ACP_ (7 casos). Inicialmente, aunque POCT_ACI_ parecía mostrar menor concordancia que POCT_ACP_, al recalcular el nivel de concordancia diagnóstica incluyendo aquellos casos que no superaban nuestra especificación, POCT_ACI_ mostró una concordancia diagnóstica levemente mayor (82,5 %) que POCT_ACP_ (81,3 %).

Dichas discrepancias diagnósticas entre POCT_ACI_ y el laboratorio central se analizaron individualmente, clasificándolas como: sin impacto clínico, impacto clínico leve (indicación de control de glucemia o repetición de la PTOG) o impacto clínico grave (indicación de tratamiento) (Tabla 4).

**Tabla 4:** Discrepancia diagnóstica entre POCT_ACI_ y el laboratorio central.

POCT_ACI_	Laboratorio central	Comentario	Impacto clínico
AGA	Normal		Sin impacto
AGA	Normal		
AGA	Normal		
AGA	Normal		
AGA	Normal		
AGA	Normal		
AGA	Normal	FQ – AIG	
Normal	ATG	FQ – AIG	
AGA	AGA+ATG	FQ	
ATG	Diabetes	FQ	
Normal	ATG		Leve
ATG	Diabetes	FQ – no se diagnosticó diabetes	
ATG	Diabetes	No se diagnosticó diabetes	
Normal	ATG		Grave
Normal	ATG		
AGA+ATG	Diabetes		
ATG	Diabetes	Repetición PTOG – no se diagnosticó diabetes	

AGA, alteración de la glucemia en ayunas; AIG, Alteración indeterminada de la glucosa; ATG, alteración de la tolerancia de glucosa; FQ, Paciente con fibrosis quística; POCT_ACI_, glucómetro con conectividad; PTOG, prueba de tolerancia oral a la glucosa.

### Costes

En la Tabla S2 del Material Suplementario se desglosan los costes asociados a los procesos de cada analizador durante el periodo de estudio. El coste global de los dispositivos POCT fue inferior al coste del laboratorio central.

### Conectividad

La tasa de errores de transcripción fue del 1,8 %. Si bien los errores de transcripción que identificamos durante el período de estudio no habrían conducido a un cambio en el diagnóstico, es importante señalar que el hecho de que no hayan tenido consecuencias en nuestro período de estudio no disminuye el riesgo potencial de que un error pueda afectar el manejo clínico de los pacientes.

Se entregaron cuestionarios a six médicos y four enfermeras, y todos recomendaron la incorporación de POCT_ACI_ en contextos clínicos similares. La puntuación media fue de 4,7 (1=peor, 5=mejor).

## Discusión

### Correlación entre las determinaciones de glucosa en las PTOG

Observamos una elevada correlación entre los glucómetros POCT y el laboratorio central, sin diferencias estadísticamente significativas, excepto en la concentración de glucosa en ayunas, donde los glucómetros POCT obtuvieron concentraciones más altas. Al revisar nuestro procedimiento en la práctica clínica, nos percatamos de que las muestras en ayunas no fueron inmediatamente centrifugadas tras la coagulación de la sangre en la Unidad de Diabetes, sino que se centrifugaron junto con las muestras a los 30 min. Las diferencias observadas se podrían explicar por la glucólisis *in vitro*, lo que podría significar que los resultados de los glucómetros en ayunas fueran más consistentes. Estudios previos han estimado que la tasa de glucólisis se encuentra entre el 5 y el 7 % (3–4 mg/dL en 30 min), lo que coincide con nuestros resultados [15]. Tras la finalización del estudio, modificamos nuestro procedimiento y, actualmente, las muestras en ayunas también se centrifugan inmediatamente tras la coagulación. Un análisis realizado seis meses después de este estudio no reveló diferencias estadísticamente significativas entre las concentraciones de glucosa en ayunas.

### Tiempo de respuesta

En numerosos contextos clínicos se ha demostrado que el uso exclusivo de dispositivos POCT reduce significativamente los TDR [7, 9, 16]. En el presente estudio, el uso del POCT_ACI_ no solo mejoró el TDR en todas determinaciones de glucosa, sino también el de las PTOG. Esto es un hallazgo relevante, dado que los resultados están disponibles de forma inmediata, lo que permitiría la toma de decisiones clínicas tras la PTOG, evitando al paciente posteriores citas médicas. Además, considerando que la transcripción de los resultados de glucosa supone unos cuatro minutos por cada PTOG, el POCT_ACI_ le habría ahorrado al personal de enfermería casi siete horas de trabajo durante el periodo de estudio.

### Concordancia diagnóstica

La PTOG en muestras de sangre venosa es el método de referencia para el diagnóstico de la diabetes y la detección de prediabetes a través de la alteración de la glucosa en ayunas (AGA) y la intolerancia a la glucosa (ITG), que son las fases intermedias entre presentar una homeostasis normal de la glucosa y el desarrollo de diabetes. No son entidades clínicas como tal, sino que indican un riesgo relativamente elevado de desarrollar diabetes y enfermedad cardiovascular, especialmente en un contexto de obesidad [3].

Dado que todas las determinaciones presentan variación analítica y biológica, al repetir la medición de un resultado alterado, se podría obtener un valor por debajo del punto de corte diagnóstico, y viceversa. Incluso teniendo una calidad analítica aceptable, una baja imprecisión analítica en torno al valor de corte diagnóstico podría desembocar en una clasificación diferente. Los clínicos deben comentar con los pacientes los signos y síntomas que éstos presentan y considerar si repetir la prueba [1].

Los resultados del presente estudio muestran una concordancia global diagnóstica aceptable, lo que coincide con estudios previos en los que se empleó sangre capilar y venosa, respectivamente [6, 17]. Sin embargo, observamos mayores discrepancias en la categoría de diabetes.

Al analizar el impacto clínico de las discrepancias diagnósticas entre POCT_ACI_ y el laboratorio central, observamos que varios de los pacientes del grupo sin impacto clínico padecían fibrosis quística. Actualmente, la PTOG es la prueba de cribado con mayor sensibilidad para detectar diabetes secundaria a fibrosis quística, a la que además de otras entidades clínicas, se añaden la tolerancia a la glucosa indeterminada (glucosa plasmática en ayunas <126 mg/dL, 2 h PTOG <140 mg/dL y PTOG ≥200 mg/dL a los 30, 60 o 90 min) [18]. En la mayoría de los pacientes, todos los analizadores diagnosticaron un trastorno indeterminado de la glucosa. Además, cabe señalar que la mayoría de las discrepancias en este grupo se correspondieron con pacientes que fueron diagnosticados de ITG por los glucómetros, con resultados normales según el laboratorio central. Dichas diferencias podrían deberse a la glucólisis *in vitro* en las muestras de sangre en ayunas. Estas reducciones en las concentraciones de glucosa podrían hacer que se pasara por alto un diagnóstico de ITG en pacientes con concentraciones de glucosa cercanas a los valores de corte. Si las muestras de sangre en ayunas se hubieran centrifugado inmediatamente, dichas discrepancias no se habrían producido.

En todos los casos con un impacto clínico leve, la discrepancia diagnóstica se produce por la proximidad de los resultados a los valores de corte. De hecho, aunque los resultados del laboratorio central cumplieran los criterios de diabetes en dos de los tres casos, únicamente se indicó control glucémico y la repetición de la PTOG.

Entre los pacientes con un impacto clínico grave, se le indicó tratamiento a un paciente cuyos resultados del laboratorio central cumplían los criterios de diabetes, aunque no se estableció un diagnóstico de dicha patología, lo que derivó en la repetición de la PTOG.

### Costes

Aunque el coste por medición suele ser superior en los dispositivos POCT, si tenemos en cuenta la totalidad de los conceptos asociados, el coste global por proceso podría ser similar o incluso inferior al del laboratorio central [7, 9, 19]. Los resultados aquí coinciden con los de estudios previos, donde evidenciamos que, mientras la medición de la glucosa tiene un mayor coste con POCT_ACI_ que en el laboratorio central, el coste global por proceso para 98 pacientes fue similar con los dos métodos. Además, estudios previos en otros contextos clínicos revelan que el uso de dispositivos POCT no solo mejoraría la eficacia clínica, sino que también son coste-efectivos [19, 20].

### Conectividad

La norma ISO 22870 específica para POCT recomienda implementar la conectividad siempre que sea posible [21]. Este es el primer estudio en comparar los resultados del laboratorio central con los obtenidos con un glucómetro conectado a una red POCT con acreditación ISO 22870. En estudios anteriores se señala que la conectividad es el método más seguro para la transferencia de datos, ya que evita errores postanalíticos relacionados con el registro manual de los datos [22, 23]. Mays et al. obtuvieron un índice de discrepancia mayor (3,7 %) relacionado con la transferencia manual de datos, que fue clínicamente relevante en 5 de cada 1.000 resultados [22]. En nuestro estudio, observamos una tasa de errores de transcripción del 1,8 %, asociados al empleo del glucómetro sin conectividad. Este hallazgo subraya la necesidad de la conectividad. En una intervención formativa dirigida a los clínicos, unida a un programa de control de calidad en línea, se logró una reducción de los errores postanalíticos [24].

Existe evidencia de la satisfacción de los clínicos con la conectividad, un factor importante a la hora de introducir un procedimiento POCT en la práctica clínica. La evidencia muestra que la satisfacción va unida a una mejor atención y mayor adherencia a las recomendaciones de manejo [25, 26]. Nuestro estudio confirma la percepción de los clínicos de que el empleo de glucómetros con conectividad presenta múltiples ventajas.

### Fortalezas y limitaciones

Nuestro estudio presenta algunas fortalezas. Aunque no existe un respaldo unánime con respecto al empleo de glucómetros para fines diagnósticos, y todavía es necesario esperar a que se publiquen las recomendaciones pertinentes en las guías de práctica clínica, este es el primer estudio en comparar los resultados obtenidos con glucómetros integrados en una red POCT con la acreditación ISO 22870, con los obtenidos en un laboratorio central con la acreditación ISO 15189 en una población pediátrica. Su mayor fortaleza es su diseño prospectivo, en el que se incluyeron tanto el glucómetro POCT_ACP_ como el POCT_ACI_. Otra fortaleza es que, en nuestro estudio, no solo se evaluó la concordancia diagnóstica, sino también su impacto clínico. Sin embargo, nuestro estudio también presenta algunas limitaciones, como el pequeño tamaño muestral y la influencia de la glucólisis *in vitro* en los resultados. Finalmente, los resultados obtenidos no se pueden extrapolar a adultos o a mujeres embarazadas.

## Conclusiones

Los glucómetros POCT muestran una elevada correlación con el laboratorio central durante la realización de la PTOG en pacientes pediátricos, ofreciendo el glucómetro con conectividad una reducción significativa del TDR, sin que ello implique un incremento de costes. La concordancia diagnóstica con el laboratorio central no ha sido la deseada, aunque la decisión clínica habría sido la misma en el 92,8 % de los casos. De este modo y, tal y como recomiendan las guías de práctica clínica, los glucómetros POCT aún no se pueden emplear para fines diagnósticos, sirviendo, de momento, como herramienta de soporte del laboratorio central durante la PTOG.

## Supplementary Material

Supplementary Material

## Material Suplementario

Este artículo incluye material suplementario (https://doi.org/10.1515/almed-2024-0017).
