# Supplementary material for: Uso de glucómetros durante la prueba de tolerancia oral a la glucosa en niños para el diagnóstico de prediabetes y diabetes. Estudio comparativo
Source: Adv Lab Med. 2024 Feb 19;5(2):197–204. [Article in Spanish] doi: 10.1515/almed-2024-0017 (PMC11206185; doi:10.1515/almed-2024-0017)
Supplement: Supplementary file 1 — Supplementary Material [file j_almed-2024-0017_suppl_001.docx]

Entregue la información solicitada en el formato y orden indicados en la plantilla. Consulte las Instrucciones de Uso que figuran a continuación.

[PLANTILLA]

| ***DECLARACIÓN*** | ***TEXTO*** |
| --- | --- |
|  |  |
| **Aprobación ética** | **Ética de la investigación La investigación relacionada con el uso humano ha cumplido con todas las regulaciones nacionales relevantes, políticas institucionales y de acuerdo con los principios de la Declaración de Helsinki y ha sido aprobada por la Junta de Revisión Institucional de los autores o un comité equivalente. Se obtuvo el consentimiento informado de todos los individuos incluidos en este estudio. El estudio fue aprobado por el Comité de Ética en Investigación Clínica de nuestro hospital (n° 4.358).** |
| **Consentimiento informado** | **Se obtuvo el consentimiento informado de todas las personas incluidas en este estudio, o de sus tutores legales o tutelados.** |
| **Contribución de los autores** | **Fabre-Estremera B, Martínez Chávez E y Manzano Ocaña M contribuyeron a la concepción y diseño del estudio y a la adquisición, análisis e interpretación de datos; Morales Sánchez MA y Pinilla Tejado participé en la adquisición de datos; En la revisión crítica participaron Carcavilla Urquí A, González-Casado I y Buño Soto A; Losantos García contribuí al análisis estadístico; Oliver P y Fernández-Calle P contribuyeron a la recopilación, análisis e interpretación de datos; revisión crítica; y supervisión del estudio. Todos los autores han aceptado la responsabilidad por el contenido completo de este manuscrito enviado y aprobado.** |
| **Conflicto de intereses** | **Los autores declaran no tener ningún conflicto de intereses.** |
| **Financiación del proyecto** | Ninguno declarado. |
| **Disponibilidad de los datos** | **No procede.** |
|  |  |
|  |  |

* No aceptamos trabajos generados por herramientas de Inteligencia Artificial (IA) o Aprendizaje Automático, principalmente porque dichas herramientas no pueden asumir la responsabilidad del trabajo presentado y, por tanto, no pueden ser consideradas como autores. Cuando dichas herramientas o tecnologías se utilicen como parte del diseño o la metodología de un estudio de investigación, su uso deberá describirse claramente en la sección de Agradecimientos.
